# Integrative Multi-Omics and Machine Learning Reveal Shared Biomarkers in Type 2 Diabetes and Atherosclerosis

**DOI:** 10.3390/ijms27010136

**Published:** 2025-12-22

**Authors:** Qingjie Wu, Zhaochu Wang, Mengzhen Fan, Linglun Hao, Jicheng Chen, Changwen Wu, Bizhen Gao

**Affiliations:** 1 Research Base of Traditional Chinese Medicine Syndrome, Fujian University of Traditional Chinese Medicine, Fuzhou 350122, China; wqj844922319@163.com (Q.W.); fanmengzhen1998@163.com (M.F.); linglunhao94@163.com (L.H.); fjtcmcjc@163.com (J.C.); 2 Graduate School, Fujian University of Traditional Chinese Medicine, Fuzhou 350122, China; 13290810160@163.com; 3 College of Integrative Medicine, Fujian University of Traditional Chinese Medicine, Fuzhou 350122, China

**Keywords:** type 2 diabetes mellitus, atherosclerosis, machine learning, immune infiltration, single-cell

## Abstract

Atherosclerosis (AS) is a leading cause of death and disability in type 2 diabetes mellitus (T2DM). However, the shared molecular mechanisms linking T2DM and atherosclerosis have not been fully elucidated. We analyzed AS- and T2DM-related gene expression profiles from the Gene Expression Omnibus (GEO) database to identify overlapping differentially expressed genes and co-expression signatures. Functional enrichment (Gene Ontology (GO)/Kyoto Encyclopedia of Genes and Genomes (KEGG)) and protein–protein interaction (PPI) network analyses were then used to describe the pathways and interaction modules associated with these shared signatures, We next applied the cytoHubba algorithm together with several machine learning methods to prioritize hub genes and evaluate their diagnostic potential and combined CIBERSORT-based immune cell infiltration analysis with single-cell RNA sequencing data to examine cell types and the expression patterns of the shared genes in specific cell populations. We identified 72 shared feature genes. Functional enrichment analysis of these genes revealed significant enrichment of inflammatory- and metabolism-related pathways. Three genes—IL1B, MMP9, and P2RY13—emerged as shared hub genes and yielded robust ANN-based predictive performance across datasets. Immune deconvolution and single-cell analyses consistently indicated inflammatory amplification and an imbalance of macrophage polarization in both conditions. Biology mapped to the hubs suggests IL1B drives inflammatory signaling, MMP9 reflects extracellular-matrix remodeling, and P2RY13 implicates cholesterol transport. Collectively, these findings indicate that T2DM and AS converge on immune and inflammatory processes with macrophage dysregulation as a central axis; IL1B, MMP9, and P2RY13 represent potential biomarkers and therapeutic targets and may influence disease progression by regulating macrophage states, supporting translational application to diagnosis and treatment of T2DM-related atherosclerosis. These findings are preliminary. Further experimental and clinical studies are needed to confirm their validity, given the limitations of the present study.

## 1. Introduction

Diabetes is a serious chronic disease. Epidemiological studies have reported that, by 2021, there were 529 million people with diabetes worldwide, among whom type 2 diabetes mellitus (T2DM) is the most common subtype, accounting for 96.0%. As one of the leading global causes of death and disability, diabetes imposes a tremendous economic burden on society [1]. Numerous studies indicate that as diabetes develops, it severely damages multiple organs and induces a series of metabolic and vascular complications (e.g., cardiovascular disease, blindness, lower-limb amputation, and renal impairment), markedly reducing quality of life [2,3,4]. Atherosclerosis (AS) is a chronic inflammatory disease of the arterial wall and arises from a complex multistep process [5]. In the diabetic state, hyperglycemia, chronic inflammation, oxidative stress, and other metabolic alterations are believed to contribute closely to the pathogenesis of atherosclerosis. Compared with the general population, individuals with diabetes more frequently exhibit calcified carotid plaques [6], and atherosclerosis in diabetes tends to occur earlier and progress more severely [7], suggesting that diabetes both triggers and exacerbates atherosclerotic disease. Large-vessel complications of diabetes—such as cardiovascular disease and lower-limb amputation—are often adverse outcomes of long-standing atherosclerosis and share substantial pathological commonalities; accumulating evidence supports atherosclerosis as a key driver of poor prognosis in diabetes [8,9].

From both pathological and clinical-outcome perspectives, T2DM and AS show striking convergence. Therefore, elucidating shared biomarkers that “bridge” these diseases is clinically meaningful for preventive interventions and improved prognosis in patients with T2DM complicated by AS. Despite abundant clinical and pathological evidence of a close association between the two, most biomarker studies have focused on candidate factors within a single disease context or on phenotype-based associations. Systematic, multilayered characterization of shared genetic and molecular regulatory features remains limited. To fill this gap, we surveyed published multi-omics datasets in GEO and applied bioinformatics and machine-learning approaches to identify key regulators within shared pathogenic pathways of T2DM and AS. These findings not only improve our understanding of regulatory networks underpinning chronic disease but may also provide valuable insights into potential biomarkers and therapeutic targets.

## 2. Results

### 2.1. WGCNA Identifies Trait-Related Modules

**Figure 1:**
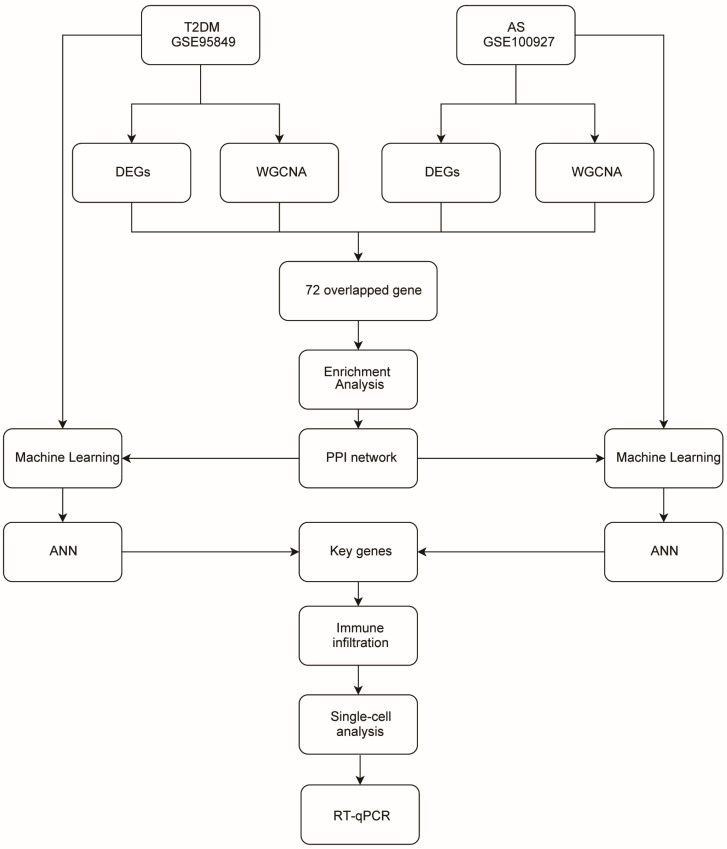
The workflow chart of this research.

**Figure 2:**
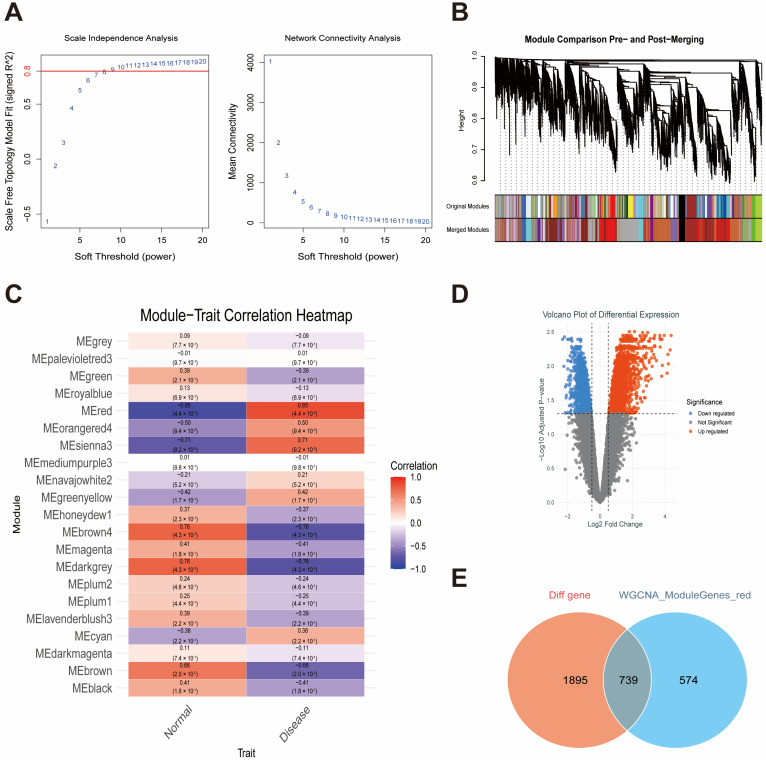
WGCNA modules and DEGs in the T2DM dataset GSE95849. (**A**) Soft-thresholding power selection. (**B**) Gene co-expression modules. (**C**) Module–trait correlations highlighting the T2DM-related module. (**D**) Volcano plot of DEGs in GSE95849. (**E**) Overlap between DEGs and T2DM-related modules. Note: In (A), the red horizontal line indicates the threshold (signed R^2^ = 0.8) used to select the soft-thresholding power (β). Abbreviations: WGCNA, weighted gene co-expression network analysis; DEG, differentially expressed gene; T2DM, type 2 diabetes mellitus.

**Figure 3:**
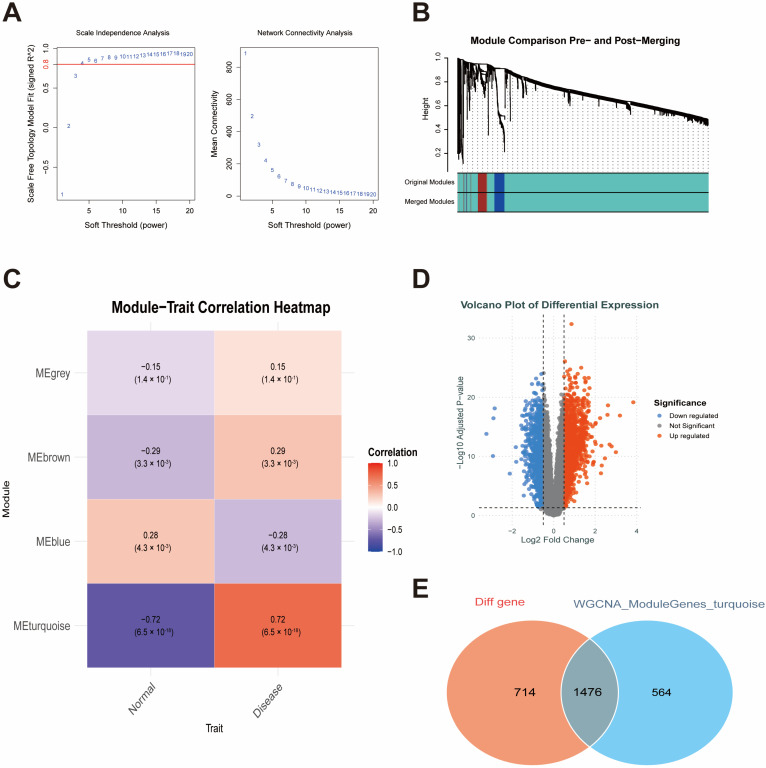
WGCNA modules and DEGs in the AS dataset GSE100927. (**A**) Soft-thresholding power selection. (**B**) Gene co-expression modules. (**C**) Module–trait correlations highlighting the AS-related module. (**D**) Volcano plot of DEGs in GSE100927. (**E**) Overlap between DEGs and AS-related modules. Note: In (A), the red horizontal line indicates the threshold (signed R^2^ = 0.8) used to select the soft-thresholding power (β). Abbreviations: WGCNA, weighted gene co-expression network analysis; DEG, differentially expressed gene; AS, atherosclerosis.

An overview of the analysis workflow is shown in Figure 1. To identify module genes associated with T2DM and AS, we applied WGCNA. In GSE95849 (T2DM), the optimal soft-thresholding power was β = 8 (Figure 2A). As the mean connectivity approached zero, the scale-free topology fit index increased. Dynamic tree cutting yielded 21 co-expression modules (Figure 2B). As shown in Figure 2C, the red module was strongly correlated with T2DM (r = 0.85), and 1313 highly connected genes within T2DM-related modules were selected as candidate module genes for downstream analyses. In GSE100927 (AS), the optimal soft-thresholding power was β = 4 (Figure 3A), producing four co-expression modules (Figure 3B). The turquoise module was significantly associated with AS (r = 0.72; Figure 3C), and 2040 highly connected genes were retained as atherosclerosis-related module genes.

### 2.2. Identification of DEGs in T2DM and AS

DEG analyses were performed for both datasets, with volcano plots for visualization. In GSE95849 (T2DM; Figure 2D), 2634 DEGs were identified. In GSE100927 (AS; Figure 3D), 2190 DEGs were identified.

### 2.3. T2DM–AS DEGs and Functional Enrichment

**Figure 4:**
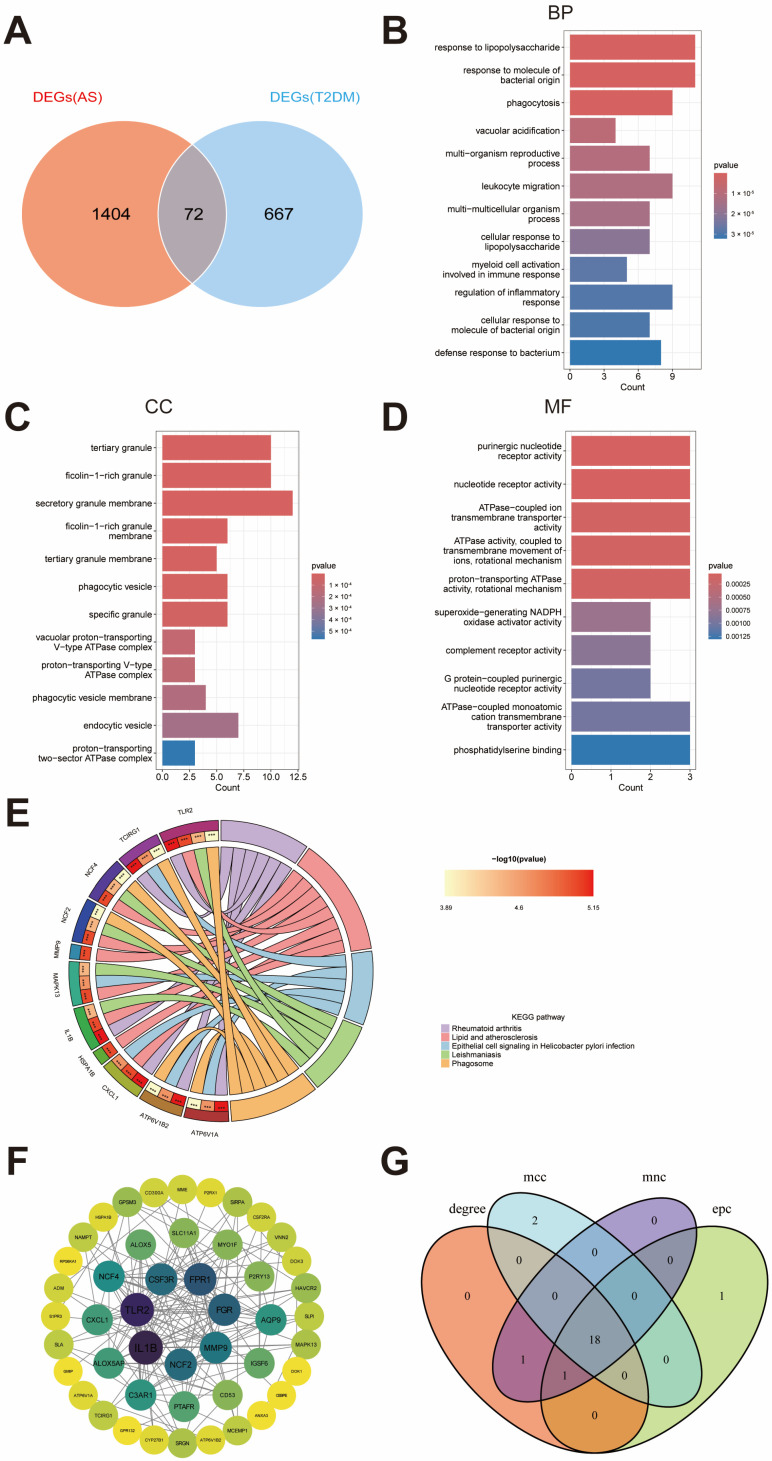
Selection and functional characterization of shared T2DM–AS genes. (**A**) Second-level intersection between T2DM (Figure 2E) and AS (Figure 3E) overlapping sets. (**B**–**D**) Gene Ontology (GO) enrichment analysis of shared genes. (**E**) Kyoto Encyclopedia of Genes and Genomes (KEGG) pathway enrichment analysis. Note: Asterisks indicate statistical significance (*** *p* < 0.001). (**F**) STRING protein–protein interaction (PPI) network of shared genes. (**G**) Intersection of top-ranked genes from four cytoHubba algorithms (MCC, MNC, Degree, EPC).

We identified genes overlapping between the DEGs and WGCNA module genes in the T2DM and AS datasets to identify potential shared markers. This analysis yielded 739 overlapping genes in T2DM (Figure 2E) and 1,476 overlapping genes in AS (Figure 3E). Intersecting these two gene sets resulted in 72 shared hub genes (Figure 4A), which were subsequently subjected to functional annotation to elucidate their biological roles. GO Biological Process terms were enriched (over-represented) for response to lipopolysaccharide, phagocytosis, leukocyte migration, myeloid cell activation, and regulation of inflammatory responses (Figure 4B). GO Cellular Component terms were enriched in tertiary granules, Flotillin-1-containing granules, secretory granule membranes, phagocytic vesicles, and the V-type ATPase complex (Figure 4C). GO Molecular Function terms were enriched for purinergic nucleotide receptor activity, ATPase-coupled transmembrane transporter activity, superoxide-generating NADPH oxidase activity, and complement receptor activity (Figure 4D). Additionally, KEGG pathway analysis identified the top five enriched pathways, including rheumatoid arthritis, lipid and atherosclerosis, epithelial signaling in Helicobacter pylori infection, leishmaniasis, and phagosome (Figure 4E).

### 2.4. PPI Network and Hub-Gene Analysis

A PPI network was constructed using STRING (Figure 4F). Four classical network-topology algorithms in cytoHubba—Maximal Clique Centrality (MCC), Maximum Neighborhood Component (MNC), Degree, and Edge Percolated Component (EPC)—were applied to rank the top 20 candidate hub genes (Table 1). Intersecting results across the four algorithms yielded 18 hub genes (Figure 4G).

### 2.5. Machine-Learning Identification and Validation of T2DM–AS Hub DEGs

**Figure 5:**
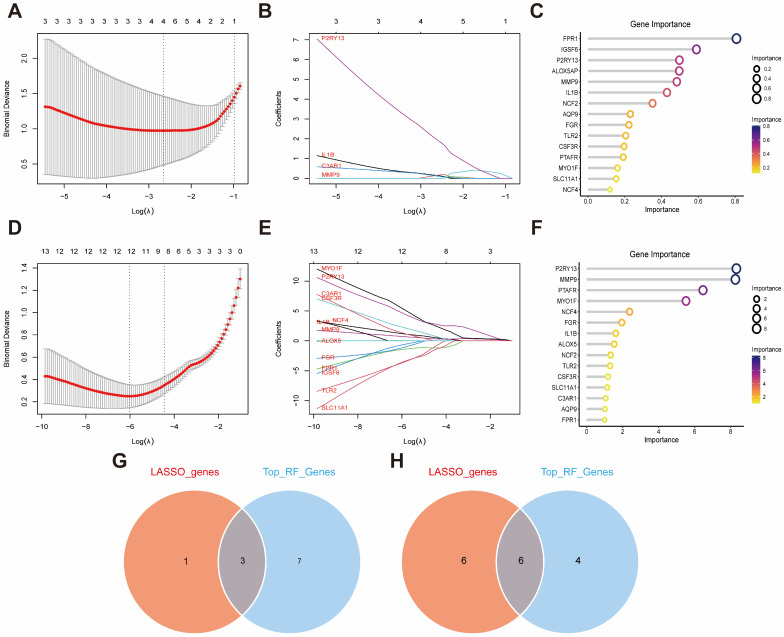
Machine-learning feature selection across cohorts and overlap of key biomarkers. (**A**,**B**) LASSO path and coefficient selection in GSE95849 (T2DM). (**C**) RF variable importance ranking in GSE95849. (**D**,**E**) LASSO path and coefficient selection in GSE100927 (AS). (**F**) RF variable importance ranking in GSE100927. (**G**,**H**) Venn overlaps of candidate genes across methods/datasets identifying shared key biomarkers in T2DM (**G**) and AS (**H**). Abbreviations: LASSO, least absolute shrinkage and selection operator; RF, random forest.

**Figure 6:**
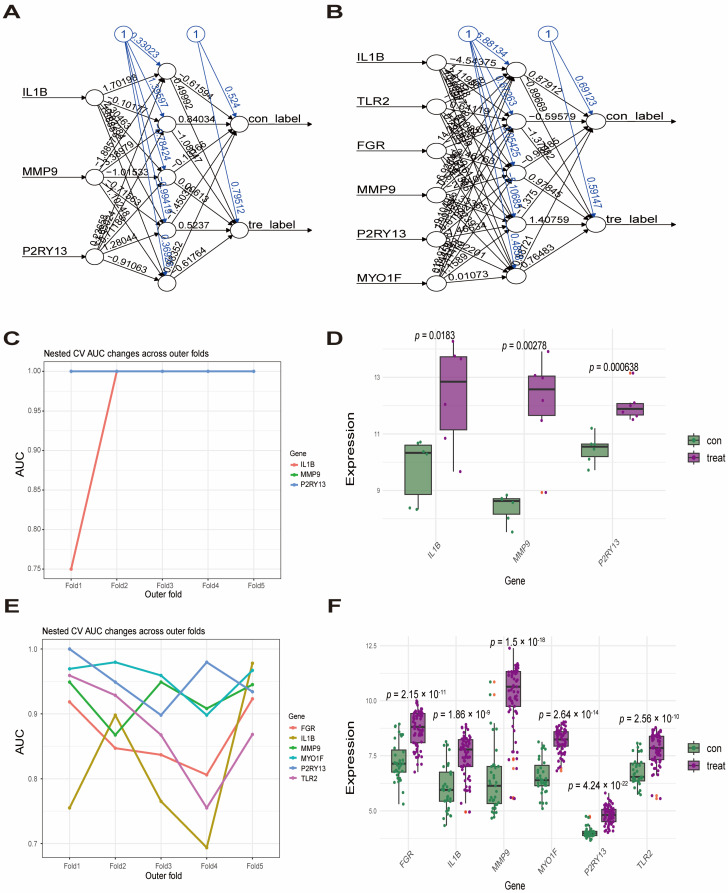
ANN-based models and expression of key genes in T2DM and AS. (**A**,**B**) Architecture of ANN models derived from prioritized genes in the T2DM (**A**) and AS (**B**) datasets. (**C**,**E**) Nested cross-validation (5-fold outer CV) AUCs for ANN models based on individual hub genes in GSE95849 (**C**) and GSE100927 (**E**). Each point represents the AUC on one outer test fold, where hyperparameters were tuned exclusively on the corresponding outer-training data via inner CV. (**D**,**F**) Differential expression of key genes in T2DM and AS datasets. Note: In (**A**,**B**), arrows show connections; blue “1” indicates bias (constant input). Abbreviations: ANN, artificial neural network; CV, cross-validation; AUC, area under the curve.

We next applied multiple machine-learning algorithms to shared comorbidity-related genes. LASSO analyses identified 4 feature genes in GSE95849 (Figure 5A,B) and 12 in GSE100927 (Figure 5D,E). RF analyses showed the top 15 most important genes in GSE95849 (Figure 5C) and likewise in GSE100927 (Figure 5F). Venn analysis selected top 10 of the most important genes in RF and intersected them with LASSO-selected genes to identify overlapping key genes: one group was IL1B, MMP9, and P2RY13, and the other group was IL1B, TLR2, FGR, MMP9, P2RY13, and MYO1F (Figure 5G,H), which ones are considered to have the greatest predictive value. ANN models were constructed based on gene weights (Figure 6A,B). In the T2DM dataset, the outer 5-fold AUC line plot (Figure 6C) showed consistently high ANN-based predictive performance for IL1B, MMP9, and P2RY13 across all outer folds. In the AS dataset, the corresponding outer-fold AUC line plot (Figure 6E) likewise indicated robust ANN-based predictive performance for IL1B, TLR2, FGR, MMP9, P2RY13, and MYO1F. All key genes were significantly differentially expressed versus controls in both datasets (Figure 6D,F).

### 2.6. Immune Infiltration and Single-Cell Results

**Figure 7:**
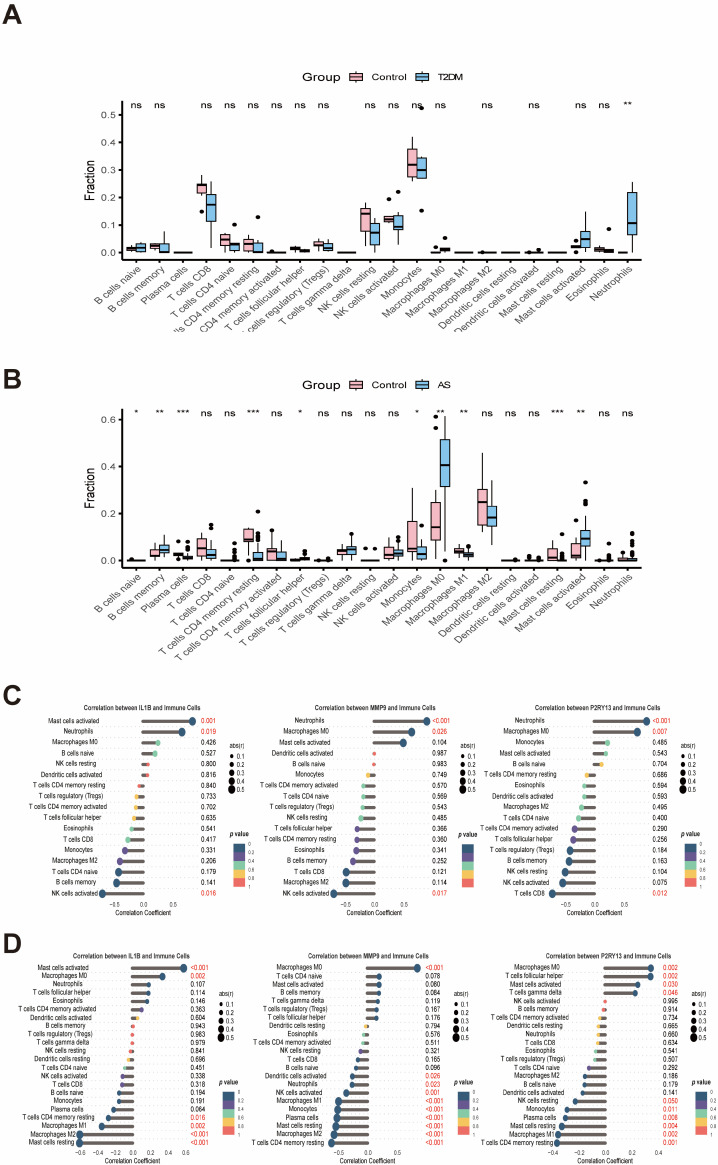
Immune-cell infiltration and associations with hub genes in T2DM and AS. Immune-cell fractions were estimated based on CIBERSORT (Cell Type Identification By Expression Resources in Silico Tools), an in silico approach for inferring cell-type proportions from bulk gene expression profiles. (**A**,**B**) Estimated fractions of immune-cell subsets in T2DM (**A**) and AS (**B**) samples. (**C**,**D**) Correlations between hub genes (IL1B, MMP9, P2RY13) and representative immune cell populations in T2DM (**C**) and AS (**D**). Statistical significance: ns, not significant; * *p* < 0.05; ** *p* < 0.01; *** *p* < 0.001.

CIBERSORT revealed altered immune infiltration patterns. In T2DM, neutrophils were increased compared with healthy controls (Figure 7A). In AS, higher proportions of memory B cells, follicular helper T cells, M0 macrophages, and activated mast cells were observed, alongside lower proportions of plasma cells, resting CD4 memory T cells, monocytes, M1 macrophages, and resting mast cells (Figure 7B). In T2DM, IL1B correlated positively with activated mast cells and neutrophils but negatively with activated NK cells; MMP9 correlated positively with neutrophils and M0 macrophages but negatively with activated NK cells; P2RY13 correlated positively with neutrophils and M0 macrophages but negatively with CD8 T cells (Figure 7C). In AS, IL1B correlated positively with activated mast cells and M0 macrophages and negatively with resting CD4 memory T cells, M1 and M2 macrophages, and resting mast cells. MMP9 correlated positively with M0 macrophages and negatively with activated dendritic cells, neutrophils, activated NK cells, M1 macrophages, monocytes, plasma cells, resting mast cells, M2 macrophages, and resting CD4 memory T cells. P2RY13 correlated positively with M0 macrophages, follicular helper T cells, activated mast cells, and γδ T cells, and negatively with resting NK cells, monocytes, plasma cells, resting mast cells, M1 macrophages, and resting CD4 memory T cells (Figure 7D).

**Figure 8:**
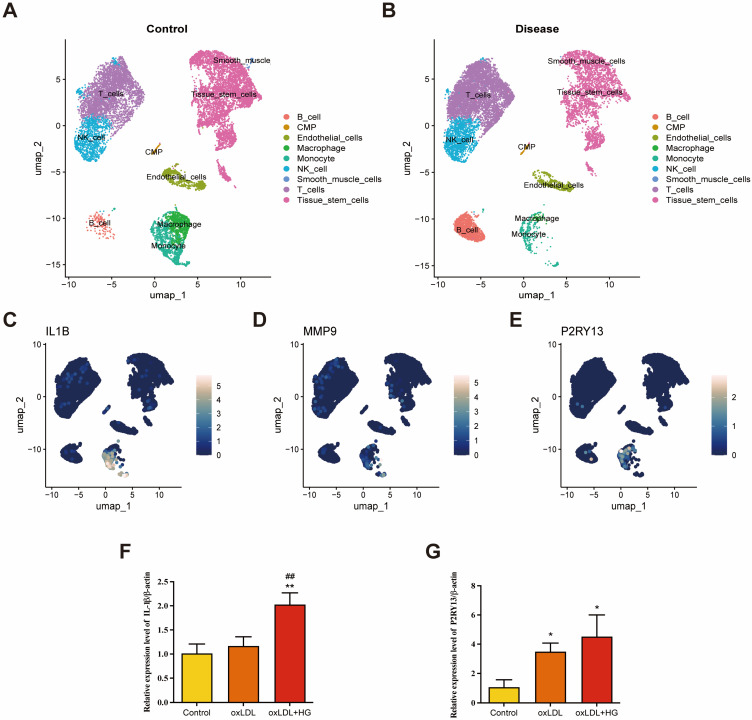
Single-cell landscape of T2DM large-vessel tissue and preliminary in vitro RT–qPCR validation of shared hub genes. (**A**,**B**) UMAP visualization and annotation of cell clusters from T2DM anterior tibial artery samples (GSE248609). (**C**–**E**) Expression patterns of IL1B, MMP9, and P2RY13 across cell populations, highlighting enrichment in macrophage/monocyte compartments. (**F**,**G**) Preliminary in vitro experiment (24 h incubation): HAEC-SV40 human aortic endothelial cells were incubated for 24 h under three conditions—control (normal glucose, 5.5 mM), oxLDL (100 μg/mL), or oxLDL + HG (30 mM glucose + 100 μg/mL oxLDL)—and IL1B (**F**) and P2RY13 (**G**) mRNA levels were quantified by RT–qPCR. * *p* < 0.05, ** *p* < 0.01 vs. the control group; ## *p* < 0.01 vs. the oxLDL group. Abbreviations: UMAP, uniform manifold approximation and projection; oxLDL, oxidized low-density lipoprotein; HG, high glucose.

Beyond transcriptomic analyses, we assessed scRNA-seq data from anterior tibial artery samples of patients with T2DM (GSE248609). After quality control (Supplementary Figures S1 and S2), UMAP clustering assigned 23,911 cells to 21 clusters (Supplementary Figure S3). Using “SingleR,” the 20 clusters were annotated into seven major cell populations, including B cells, common myeloid progenitors (CMP), endothelial cells, macrophages, monocytes, and NK cells (Figure 8A). FeaturePlot mapping showed broad distribution of IL1B and MMP9 across multiple cell types, whereas P2RY13 localized primarily to macrophage and monocyte clusters (Figure 8C–E). It is worth noting that these three shared genes are highly enriched in both the macrophage and monocyte chambers. Overall, the observed immune-cell pattern indicates that in AS lesions, there exists an expanded population of unpolarized M0 macrophages alongside diminished M1 and M2 subsets. This finding suggests impaired dynamics of macrophage polarization and a transition towards an inflammatory microenvironment that is less effective in resolving tissue injury along the T2DM–AS axis.

### 2.7. Evaluating the Expression of the Candidate Genes in the HAEC-SV40 Cell Line

To validate the expression changes of key genes in an oxLDL/HG-induced endothelial injury model, RT–qPCR was performed in HAEC-SV40 human aortic endothelial cells. IL1B was significantly upregulated in the oxLDL + HG group compared with both the control and oxLDL groups, whereas no significant difference was observed between the control and oxLDL groups (Figure 8F). P2RY13 was significantly increased in both the oxLDL and oxLDL + HG groups versus control, suggesting a greater responsiveness to oxLDL stimulation (Figure 8G). MMP9 was not consistently detectable under these conditions, likely due to low basal expression and remaining below the detection limit after 24 h of oxLDL ± HG treatment; therefore, it was excluded from quantitative analysis.

## 3. Discussion

Atherosclerosis is a leading cause of disability and mortality among patients with type 2 diabetes mellitus, imposing a major public health burden. Early detection and timely intervention are widely regarded as critical for reducing complications and improving prognosis. In this study, we integrated multiple omics datasets and combined bioinformatics with machine learning methods to fully explore the pathological association between T2DM and AS.

Across the two diseases, we identified 72 overlapping genes. GO and KEGG enrichment analyses revealed significant over-representation of inflammatory and immune pathways, delineating a landscape of innate immune activation and inflammatory amplification that jointly contribute to the onset and progression of AS and T2DM. Complement engagement and oxidative stress were also enriched and are known to play pivotal roles in plaque initiation, progression, and repair [10,11].

Machine-learning approaches have been widely adopted in medicine, where supervised and unsupervised models learn and validate task-relevant features to markedly enhance diagnostic efficiency [11,12]. In our work, LASSO regression and RF algorithms prioritized three reliable shared genes—IL1B, MMP9, and P2RY13—across both diseases. ROC analyses further substantiated their robustness.

Endothelial dysfunction is a defining feature of vascular disease, and a pro-inflammatory milieu is a principal driver. IL1B is a prototypical, potent pro-inflammatory cytokine. Prior studies show that hyperglycemia augments pro-inflammatory cytokine secretion and that IL1B is a key driver of chronic low-grade inflammation in diabetes [13,14]. The expression of adhesion molecules such as ICAM-1 and VCAM-1 mediates leukocyte recruitment to endothelium. IL1B induces these adhesion molecules [15]. Randomized controlled trials have shown that therapeutic inhibition of IL1B can significantly reduce adverse cardiovascular events compared with placebo [16]. Conversion of inactive pro-IL1B into active IL1B is largely mediated by infiltrating neutrophils [17]. Consistent with this, our immune-infiltration analysis in T2DM datasets showed neutrophil upregulation. Prior literature also reports that neutrophil extracellular traps activate the inflammasome and destabilize plaques [18]. The clinical detection of IL1B is mainly carried out through qRT-PCR (mRNA) and ELISA/digital immunoassay (protein). There are already mature clinical drugs for the treatment targeting IL1B (such as Anakinra and Canakinumab), and extensive research has been conducted in various inflammatory diseases, cardiovascular diseases, and tumor prevention [19,20]. Together, these observations support an amplified IL1B–neutrophil inflammatory axis and suggest that the chronic inflammatory state sculpted by T2DM actively drives AS pathology.

Vascular remodeling is a central pathological mechanism underlying the progression of multiple vascular disorders, including hypertension, aneurysm, atherosclerosis, and venous over-dilation. Matrix metalloproteinase-9 (MMP9) is a secreted zinc-dependent metallopeptidase with proteolytic activity that degrades and remodels the extracellular matrix (ECM), regulating the turnover of collagen and elastin. MMP9 also releases or activates numerous bioactive mediators (growth factors, chemokines, cytokines), thereby influencing tissue remodeling, immune responses, and angiogenesis [21,22]. Accumulating evidence indicates that ECM remodeling reduces arterial elasticity and compliance, leading to vascular stiffening [23]. MMP9 can remodel the arterial wall and promote cardiovascular remodeling, thereby facilitating plaque formation [24]. In diabetes, hyperglycemia enhances MMP9 secretion from large-vessel endothelial cells [25]. A recent study spanning in vitro systems, diabetic mouse models, and newly diagnosed T2DM cohorts demonstrated that MMP9 has potential for early identification of carotid and coronary plaques [26]. Moreover, MMP9 polymorphisms are associated with genetic susceptibility to cardiovascular disease among individuals with T2DM [27]. An early clinical prospective study demonstrated that plasma MMP9 concentration was associated with cardiovascular mortality in patients with coronary arteries and could serve as a predictor [28]. A literature review indicates that the detection methods for MMP9 are relatively mature. Besides the traditional ELISA/immunohistochemistry and gelatin enzyme spectrometry methods, new nanosensors and biosensors can also be used for the detection of MMP9. In terms of treatment, a wide range of MMP family inhibitors and multiple generations of MMP9 inhibitors (small molecules, proteins/antibodies, etc.) have been studied in animal experiments and some clinical trials. For instance, the extensive inhibition of MMP by doxycycline has been explored in clinical trials as a means to regulate stromal degradation in patients with lower abdominal aortic aneurysms [29,30].

P2RY13 is a G-protein-coupled receptor responsive to extracellular purine and pyrimidine nucleotides that negatively regulates adenylyl cyclase; it plays a crucial role in inflammatory and immune imbalance [31,32,33,34]. Compared with wild-type mice, P2RY13-deficient mice exhibit greater susceptibility to diet-induced insulin resistance [35]. P2RY13 signaling regulates high-density lipoprotein (HDL) endocytosis and promotes HDL uptake, thereby mediating reverse cholesterol transport to the liver; P2RY13 deficiency in mice reduces hepatic HDL-cholesterol uptake, content, and output, compromising HDL’s atheroprotective function and increasing AS risk [36,37,38,39]. Clinically, higher P2RY13 expression has been associated with vascular protection [40]. These findings suggest that P2RY13 expression or activation is closely linked to metabolic homeostasis; reduced expression may impair HDL-mediated lipid clearance, fostering foam-cell formation and lipid deposition. Unlike IL1B and MMP9, P2RY13 currently lacks approved targeted therapies or routine clinical assays. Small-molecule agonists and antagonists of P2RY13 remain at the preclinical stage, so our data primarily support P2RY13 as an exploratory drug target and mechanistic link between metabolic dysregulation and plaque biology in T2DM-related atherosclerosis.

Beyond the bioinformatics results, our endothelial cell experiment provides some orthogonal support for the involvement of IL1B and P2RY13 in T2DM-related atherosclerotic injury. In HAEC-SV40 cells, IL1B was markedly induced only under combined oxLDL plus high-glucose conditions, which is in line with the idea that IL1B reflects a more “diabetic–atherogenic” inflammatory milieu rather than a response to lipid loading alone. P2RY13 expression increased in both oxLDL and oxLDL + high-glucose groups, suggesting that this purinergic receptor is sensitive to oxLDL-triggered stress in vascular endothelium and may act at the interface between nucleotide signaling and lipid handling. By contrast, MMP9 mRNA was barely detectable in this model, which may reflect the fact that, in atherosclerotic lesions, MMP9 is more abundantly expressed in macrophages and other matrix-remodeling cell types than in endothelial cells, and may require more appropriate models or stronger inflammatory stimulation to be robustly induced. We therefore view the qRT-PCR data as a modest but supportive piece of evidence: they are consistent with our bioinformatics results for IL1B and P2RY13, while at the same time highlighting potential cell type- and context-dependence of MMP9 biology. Taken together, these preliminary in vitro data to some extent strengthen the biological plausibility of IL1B and P2RY13 as shared T2DM–AS-related markers and help to frame more focused mechanistic and translational studies in future work.

Compared with bulk immune-infiltration analyses, scRNA-seq offers superior resolution for mapping hub-gene localization across cell clusters and for strengthening gene–cell evidence chains. By integrating CIBERSORT and scRNA-seq, our multi-omics analysis delineates a macrophage-centered immune-remodeling landscape shared by T2DM and AS: in both disease datasets, macrophages/monocyte-derived cells emerge as key cellular sources. Notably, hub genes generally show negative correlations with M1 and M2 macrophage signatures, implying a bias toward an unpolarized, homeostasis-like (M0-like) state that disrupts macrophage polarization and fosters disease progression. M2 macrophages are responsible for efferocytosis and debris clearance and play critical roles in tissue repair and fibrosis [41]. In ApoE-deficient mice, ionizing irradiation that promotes macrophage polarization reduces IL1B expression and confers atheroprotection [42]. Experimentally, driving plaque-infiltrating macrophages toward an M2 phenotype stabilizes atherosclerotic lesions [43]. These observations align with our findings, suggesting that inefficient efferocytosis and repair sustain local inflammation and enlarge the necrotic core in T2DM-complicated AS. In this context, the CIBERSORT profile of increased M0 and decreased M1/M2 macrophage signatures in our study may reflect a macrophage pool trapped in an incompletely polarized state, which fails to fully engage either inflammatory clearance or pro-resolving programs and thereby favors progression toward unstable plaques in patients with T2DM. In line with this macrophage-centered pattern, the scRNA-seq localization indicates that IL1B and MMP9 function as broadly distributed inflammatory and matrix-remodeling mediators, whereas macrophage/monocyte-enriched P2RY13 links purinergic signaling to lipid handling within the atherosclerotic plaque in T2DM–AS.

This study has limitations. First, the discovery datasets from GEO have modest sample sizes, particularly GSE95849 with only 6 T2DM patients and 6 controls, which inevitably reduces statistical power and may limit the generalizability of our findings. To partly mitigate this, we combined differential expression analysis with multiple machine-learning approaches and nested cross-validation, and further confirmed the expression trends of hub genes by qPCR in relevant cell models. Nevertheless, the experimental validation remains restricted to RNA-level measurements in a single human aortic endothelial cell line, and future studies should extend these findings to protein-level assays and additional vascular and immune cell types. In particular, MMP9 may need to be examined in macrophages or other matrix-remodeling cells, as well as in more complex in vitro or in vivo models, to better capture its context-dependent role in atherosclerotic remodeling. Secondly, this work should be regarded as an exploratory study that provides initial insight into shared molecular mechanisms between T2DM and AS, and the findings will need to be externally validated in larger, well-designed clinical cohorts to establish their clinical relevance and prognostic value.

## 4. Materials and Methods

### 4.1. Data Sources

Datasets related to T2DM and atherosclerotic lesions were retrieved from GEO “http://www.ncbi.nlm.nih.gov/geo/ (accessed on 18 June 2025)”. GSE95849 (GPL22448) includes 6 patients with T2DM and 6 controls [44]. GSE100927 (GPL17077) includes 69 patients with atherosclerotic lesions and 35 controls [45]. GSE248609 is a high-throughput scRNA-seq dataset derived from anterior tibial artery specimens from patients with T2DM [46]. The analysis workflow is shown in Figure 1.

### 4.2. Weighted Gene Co-Expression Network Analysis (WGCNA)

We use the R package “WGCNA” (version 1.73) to identify gene modules that are significantly associated with the clinical features of T2DM and AS. The function “pickSoftThreshold” was applied to select an appropriate soft-thresholding power (β). An adjacency matrix was constructed and transformed into a topological overlap matrix (TOM). Genes with similar expression profiles were clustered using average linkage hierarchical clustering based on TOM-derived dissimilarity, and assigned to modules via dynamic tree cutting. Finally, we computed correlations between module eigengenes and clinical traits.

### 4.3. Identification of DEGs

DEGs in the T2DM dataset GSE95849 and the AS dataset GSE100927 were screened with the R package “limma” (version 3.64.1). Thresholds were set at adjusted *p*-value < 0.05 and |log2 fold change| > 0.5.

### 4.4. Functional Enrichment Analysis

GO enrichment (biological process, cellular component, and molecular function) and KEGG pathway analyses were performed using the R package “clusterProfiler” (version 4.16.0).

### 4.5. PPI Network Analysis

We used STRING to explore PPI among the identified genes and visualized networks in Cytoscape (version 3.10.3). Network topology was evaluated with Cytoscape’s NetworkAnalyzer. Hub genes were prioritized using the cytoHubba plugin.

### 4.6. Machine Learning and Clinical-Feature Analysis

Least absolute shrinkage and selection operator (LASSO) regression with L1 regularization was implemented via “glmnet” (version 4.1-9). For LASSO, we applied 10-fold cross-validation to select the optimal penalty parameter λ and then extracted genes with non-zero coefficients at this λ. Random forest (RF) was conducted with the “randomForest” (version 4.7-1.2) package. For RF, we relied on the built-in out-of-bag (OOB) error estimates and MeanDecreaseGini to rank genes and select top candidates. ANN-based models were built using “neuralnet” (version 1.44.2) and “NeuralNetTools” (version 1.5.3) in R to nominate key genes relevant to both diseases. Additionally, for the evaluation of ANN-based predictive performance of the candidate biomarkers, we used a 5-fold outer CV stratified by case/control status, with a 3-fold inner CV within each outer-training set to tune the number of hidden neurons and other hyperparameters, and summarized predictive performance using outer-fold AUCs and pooled ROC curves.

### 4.7. Immune Infiltration and Single-Cell Analyses

CIBERSORT was applied to estimate relative proportions of 22 infiltrating immune-cell subsets from bulk gene-expression profiles. The Wilcoxon test was used to compare the differences in the proportion of 22 types of immune cells between T2DM and AS samples and healthy control samples, respectively; a *p*-value < 0.05 was considered statistically significant. Spearman correlation analysis was used to assess associations between key genes and specific immune-cell subsets. For the T2DM large-vessel scRNA-seq dataset (GSE248609), data were processed in “Seurat” (version 5.3.0) to build a Seurat object. After quality control and filtering, the top 2000 highly variable genes were used for principal components analysis (PCA). Unsupervised clustering was performed with UMAP for visualization of cellular substructure. The “FeaturePlot” function was used to explore marker expression across clusters, and “SingleR” (version 2.10.0) provided biological annotations for each cluster.

### 4.8. Cell Culture and Treatment

The human aortic endothelial cell line HAEC-SV40 was obtained from SUNNCELL Biotechnology (Wuhan, China). Cells were cultured in H-DMEM supplemented with 10% FBS and 1% penicillin/streptomycin at 37 °C in a humidified incubator with 5% CO_2_. After one PBS wash, cells were treated for 24 h as follows: (1) control (normal glucose, 5.5 mM); (2) oxLDL (100 μg/mL; Human Ox-LDL, MKBio, Shanghai, China; #MP6009); and (3) oxLDL + high glucose (HG; 30 mM glucose + 100 μg/mL oxLDL), with HG achieved by adding D-glucose (Sigma-Aldrich, St. Louis, MO, USA; #D9434). Cells were then harvested and collected as pellets for subsequent experiments.

### 4.9. RNA Extraction and Quantitative Real-Time PCR Analysis

Total RNA was isolated from cells using TRIzon Total RNA Extraction Reagent (CWBIO, Taizhou, China; #CW0580S) with chloroform (Merck, Darmstadt, Germany; #C2432) and quantified; RNA was stored at −80 °C until use. cDNA was generated using the SuperRT III One-Step RT Kit with gDNA Remover (Biosharp, Beijing, China; #BL1020B). qPCR was performed in 20 μL reactions using UltraSYBR Mixture (CWBIO, Taizhou, China; #CW0957) on a real-time PCR system. β-actin served as the endogenous control, and relative mRNA expression was calculated using the 2^−ΔΔCt^ method. Primer sequences were as follows: β-actin (F: GAGAAAATCTGGCACCACACC; R: GGATAGCACAGCCTGGATAGCAA), IL1β (F: GCTTATTACAGTGGCAATGAGGAT; R: TAGTGGTGGTCGGAGATTCG), MMP9 (F: TTGACAGCGACAAGAAGTGG; R: CCTCAGTGAAGCGGTACATAG), and P2RY13 (F: CTGGGGCTGAAATGGCATCA; R: CACACAAAGAAGACAGCCACG).

### 4.10. Statistical Analysis

All statistical analyses were performed using R (version 4.5.1) and GraphPad Prism (version 10.6.1). Two-group comparisons were conducted using the *t*-test, and multiple-group comparisons were performed using one-way ANOVA. A *p*-value < 0.05 was considered statistically significant.

## 5. Conclusions

We identified three candidate biomarkers for T2DM-related atherosclerosis—IL1B, MMP9, and P2RY13. These biomarkers not only support the construction of predictive models for T2DM-associated AS but also participate in inflammatory and immune pathways underlying disease progression. We further discovered that these shared biomarkers might be involved in regulating macrophage polarization imbalance in T2DM complicated by AS. These findings may clarify new targets for T2DM-related atherosclerosis and advance diagnostic and therapeutic strategies. Our conclusions should be interpreted as preliminary. Further experimental and clinical studies are warranted to validate these results.

## Figures and Tables

**Table 1:** Hub Genes Identified by Multiple PPI Topological Algorithms.

Gene	Description	MCC	MNC	Degree	EPC
ALOX5	arachidonate 5-lipoxygenase	5880	9	9	19.533
ALOX5AP	arachidonate 5-lipoxygenase-activating protein	10,114	11	11	20.409
AQP9	aquaporin 9	1664	12	12	20.771
C3AR1	complement component 3a receptor 1	422	12	12	20.26
CSF3R	colony-stimulating factor 3 receptor (granulocyte)	7514	14	16	22.092
CXCL1	chemokine (C-X-C motif) ligand 1 (melanoma growth-stimulating activity, alpha)	262	11	11	19.792
FGR	FGR proto-oncogene, Src family tyrosine kinase	11,741	16	17	21.855
FPR1	formyl peptide receptor 1	13,618	18	18	22.985
IGSF6	immunoglobulin superfamily, member 6	104	9	9	18.533
IL1B	interleukin 1, beta	13,641	24	25	22.966
MMP9	matrix metallopeptidase 9	1614	15	15	21.094
MYO1F	myosin IF	29	6	7	15.35
NCF2	neutrophil cytosolic factor 2	12,754	16	16	22.511
NCF4	neutrophil cytosolic factor 4, 40kDa	11,575	12	13	21.048
P2RY13	purinergic receptor P2Y, G-protein coupled, 13	33	6	7	16.029
PTAFR	platelet-activating factor receptor	151	9	10	19.155
SLC11A1	solute carrier family 11 (proton-coupled divalent metal ion transporter), member 1	722	7	7	17.813
TLR2	toll-like receptor 2	13,724	22	22	22.831

## Data Availability

The data presented in this study are openly available in GEO “https://www.ncbi.nlm.nih.gov/geo/ (accessed on 18 June 2025)”.

## Supplementary Materials

The following supporting information can be downloaded at https://www.mdpi.com/article/10.3390/ijms27010136/s1.

## Abbreviations

The following abbreviations are used in this manuscript:

T2DM	Type 2 diabetes mellitus
AS	Atherosclerosis
DEGs	Differentially expressed genes
WGCNA	Weighted Gene Co-expression Network Analysis
GEO	Gene Expression Omnibus
GO	Gene Ontology
KEGG	Kyoto Encyclopedia of Genes and Genomes
LASSO	Least absolute shrinkage and selection operator
RF	Random forest
ANN	Artificial neural network
ROC	Receiver operating characteristic
IL1B	Interleukin 1, beta
MMP9	Matrix metallopeptidase 9
P2RY13	Purinergic receptor P2Y, G-protein coupled, 13
OOB	Out-of-bag
CV	Cross-validation

## References

[1] Ong K.L., Stafford L.K., McLaughlin S.A., Boyko E.J., Vollset S.E., Smith A.E., Dalton B.E., Duprey J., Cruz J.A., Hagins H. (2023). Global, Regional, and National Burden of Diabetes from 1990 to 2021, with Projections of Prevalence to 2050: A Systematic Analysis for the Global Burden of Disease Study 2021. Lancet.

[2] Tinajero M.G., Malik V.S. (2021). An Update on the Epidemiology of Type 2 Diabetes: A Global Perspective. Endocrinol. Metab. Clin. N. Am..

[3] Avogaro A., Fadini G.P. (2019). Microvascular Complications in Diabetes: A Growing Concern for Cardiologists. Int. J. Cardiol..

[4] Demir S., Nawroth P.P., Herzig S., Ekim Üstünel B. (2021). Emerging Targets in Type 2 Diabetes and Diabetic Complications. Adv. Sci..

[5] Falk E. (2006). Pathogenesis of Atherosclerosis. J. Am. Coll. Cardiol..

[6] Differences in Carotid Plaques Between Symptomatic Patients with and Without Diabetes Mellitus. https://www.ahajournals.org/doi/epub/10.1161/ATVBAHA.118.312092.

[7] Patsouras A., Farmaki P., Garmpi A., Damaskos C., Garmpis N., Mantas D., Diamantis E. (2019). Screening and Risk Assessment of Coronary Artery Disease in Patients with Type 2 Diabetes: An Updated Review. In Vivo.

[8] Kaplovitch E., Eikelboom J.W., Dyal L., Aboyans V., Abola M.T., Verhamme P., Avezum A., Fox K.A.A., Berkowitz S.D., Bangdiwala S.I. (2021). Rivaroxaban and Aspirin in Patients with Symptomatic Lower Extremity Peripheral Artery Disease. JAMA Cardiol..

[9] Li Y., Liu Y., Liu S., Gao M., Wang W., Chen K., Huang L., Liu Y. (2023). Diabetic Vascular Diseases: Molecular Mechanisms and Therapeutic Strategies. Signal Transduct. Target. Ther..

[10] Maffia P., Mauro C., Case A., Kemper C. (2024). Canonical and Non-Canonical Roles of Complement in Atherosclerosis. Nat. Rev. Cardiol..

[11] Poznyak A.V., Grechko A.V., Orekhova V.A., Khotina V., Ivanova E.A., Orekhov A.N. (2020). NADPH Oxidases and Their Role in Atherosclerosis. Biomedicines.

[12] Haug C.J., Drazen J.M. (2023). Artificial Intelligence and Machine Learning in Clinical Medicine, 2023. N. Engl. J. Med..

[13] Dinarello C.A., Donath M.Y., Mandrup-Poulsen T. (2010). Role of IL-1β in Type 2 Diabetes. Curr. Opin. Endocrinol. Diabetes Obes..

[14] Peiró C., Lorenzo Ó., Carraro R., Sánchez-Ferrer C.F. (2017). IL-1β Inhibition in Cardiovascular Complications Associated to Diabetes Mellitus. Front. Pharmacol..

[15] Shikama Y., Aki N., Hata A., Nishimura M., Oyadomari S., Funaki M. (2015). Palmitate-Stimulated Monocytes Induce Adhesion Molecule Expression in Endothelial Cells via IL-1 Signaling Pathway. J. Cell. Physiol..

[16] Everett B.M., MacFadyen J.G., Thuren T., Libby P., Glynn R.J., Ridker P.M. (2020). Inhibition of Interleukin-1β and Reduction in Atherothrombotic Cardiovascular Events in the CANTOS Trial. J. Am. Coll. Cardiol..

[17] Dinarello C.A. (2011). A Clinical Perspective of IL-1β as the Gatekeeper of Inflammation. Eur. J. Immunol..

[18] Shetty S., Subramanian M. (2025). Neutrophil Extracellular Traps (NETs) as Drivers of Atherosclerosis: Pathogenic Mechanisms and Therapeutic Opportunities. Pharmacol. Ther..

[19] Mai W., Liao Y. (2020). Targeting IL-1β in the Treatment of Atherosclerosis. Front. Immunol..

[20] Wijdan S.A., Bokhari S.M.N.A., Alvares J., Latif V. (2025). The Role of Interleukin-1 Beta in Inflammation and the Potential of Immune-Targeted Therapies. Pharmacol. Res.—Rep..

[21] Mittal R., Patel A.P., Debs L.H., Nguyen D., Patel K., Grati M., Mittal J., Yan D., Chapagain P., Liu X.Z. (2016). Intricate Functions of Matrix Metalloproteinases in Physiological and Pathological Conditions. J. Cell. Physiol..

[22] Wang X., Khalil R.A. (2018). Matrix Metalloproteinases, Vascular Remodeling, and Vascular Disease. Adv. Pharmacol..

[23] Wang M., Kim S.H., Monticone R.E., Lakatta E.G. (2015). Matrix Metalloproteinases Promote Arterial Remodeling in Aging, Hypertension, and Atherosclerosis. Hypertension.

[24] Wågsäter D., Zhu C., Björkegren J., Skogsberg J., Eriksson P. (2011). MMP-2 and MMP-9 Are Prominent Matrix Metalloproteinases during Atherosclerosis Development in the Ldlr-/-Apob100/100 Mouse. Int. J. Mol. Med..

[25] Ghandour F., Kassem S., Simanovich E., Rahat M.A. (2024). Glucose Promotes EMMPRIN/CD147 and the Secretion of Pro-Angiogenic Factors in a Co-Culture System of Endothelial Cells and Monocytes. Biomedicines.

[26] Liu B., Su L., Loo S.J., Gao Y., Khin E., Kong X., Dalan R., Su X., Lee K.-O., Ma J. (2024). Matrix Metallopeptidase 9 Contributes to the Beginning of Plaque and Is a Potential Biomarker for the Early Identification of Atherosclerosis in Asymptomatic Patients with Diabetes. Front. Endocrinol..

[27] Buraczynska M., Wrzos S., Zaluska W. (2023). MMP9 Gene Polymorphism (Rs3918242) Increases the Risk of Cardiovascular Disease in Type 2 Diabetes Patients. J. Clin. Med..

[28] Blankenberg S., Rupprecht H.J., Poirier O., Bickel C., Smieja M., Hafner G., Meyer J., Cambien F., Tiret L. (2003). Plasma Concentrations and Genetic Variation of Matrix Metalloproteinase 9 and Prognosis of Patients With Cardiovascular Disease. Circulation.

[29] Maguire E.M., Pearce S.W.A., Xiao R., Oo A.Y., Xiao Q. (2019). Matrix Metalloproteinase in Abdominal Aortic Aneurysm and Aortic Dissection. Pharmaceuticals.

[30] Abdul-Hussien H., Hanemaaijer R., Verheijen J.H., van Bockel J.H., Geelkerken R.H., Lindeman J.H.N. (2009). Doxycycline Therapy for Abdominal Aneurysm: Improved Proteolytic Balance through Reduced Neutrophil Content. J. Vasc. Surg..

[31] Pérez-Sen R., Gómez-Villafuertes R., Ortega F., Gualix J., Delicado E.G., Miras-Portugal M.T., Atassi M.Z. (2017). An Update on P2Y13 Receptor Signalling and Function. Protein Reviews.

[32] Wu X., Wei S., Chen M., Li J., Wei Y., Zhang J., Dong W. (2022). P2RY13 Exacerbates Intestinal Inflammation by Damaging the Intestinal Mucosal Barrier via Activating IL-6/STAT3 Pathway. Int. J. Biol. Sci..

[33] Yin S., Yang X., Li H., Li C., Li C., Chen C., Ye S., Zou L., Liang S., Liu S. (2024). P2Y13 Receptor Involved in HIV-1 Gp120 Induced Neuropathy in Superior Cervical Ganglia through NLRP3 Inflammasome Activation. Neuropharmacology.

[34] Werder R.B., Ullah M.A., Rahman M.M., Simpson J., Lynch J.P., Collinson N., Rittchen S., Rashid R.B., Sikder M.A.A., Handoko H.Y. (2022). Targeting the P2Y13 Receptor Suppresses IL-33 and HMGB1 Release and Ameliorates Experimental Asthma. Am. J. Respir. Crit. Care Med..

[35] Duparc T., Gore E., Combes G., Beuzelin D., Pires Da Silva J., Bouguetoch V., Marquès M.-A., Velazquez A., Viguerie N., Tavernier G. (2024). P2Y13 Receptor Deficiency Favors Adipose Tissue Lipolysis and Worsens Insulin Resistance and Fatty Liver Disease. JCI Insight.

[36] Fabre A.C., Malaval C., Ben Addi A., Verdier C., Pons V., Serhan N., Lichtenstein L., Combes G., Huby T., Briand F. (2010). P2Y13 Receptor Is Critical for Reverse Cholesterol Transport. Hepatology.

[37] Lichtenstein L., Serhan N., Annema W., Combes G., Robaye B., Boeynaems J.-M., Perret B., Tietge U.J.F., Laffargue M., Martinez L.O. (2013). Lack of P2Y13 in Mice Fed a High Cholesterol Diet Results in Decreased Hepatic Cholesterol Content, Biliary Lipid Secretion and Reverse Cholesterol Transport. Nutr. Metab..

[38] Lichtenstein L., Serhan N., Espinosa-Delgado S., Fabre A., Annema W., Tietge U.J.F., Robaye B., Boeynaems J.-M., Laffargue M., Perret B. (2015). Increased Atherosclerosis in P2Y13/Apolipoprotein E Double-Knockout Mice: Contribution of P2Y13 to Reverse Cholesterol Transport. Cardiovasc. Res..

[39] Pons V., Serhan N., Gayral S., Malaval C., Nauze M., Malet N., Laffargue M., Galés C., Martinez L.O. (2013). Role of the Ubiquitin–Proteasome System in the Regulation of P2Y13 Receptor Expression: Impact on Hepatic HDL Uptake. Cell. Mol. Life Sci..

[40] Jiao T., Collado A., Mahdi A., Jurga J., Tengbom J., Saleh N., Verouhis D., Böhm F., Zhou Z., Yang J. (2022). Erythrocytes from Patients with ST-Elevation Myocardial Infarction Induce Cardioprotection through the Purinergic P2Y13 Receptor and Nitric Oxide Signaling. Basic Res. Cardiol..

[41] Lafuse W.P., Wozniak D.J., Rajaram M.V.S. (2020). Role of Cardiac Macrophages on Cardiac Inflammation, Fibrosis and Tissue Repair. Cells.

[42] Rey N., Ebrahimian T., Gloaguen C., Kereselidze D., Christelle E., Brizais C., Bachelot F., Riazi G., Monceau V., Demarquay C. (2024). Low to Moderate Dose 137Cs (γ) Radiation Promotes M2 Type Macrophage Skewing and Reduces Atherosclerotic Plaque CD68+ Cell Content in ApoE(−/−) Mice. Sci. Rep..

[43] Khallou-Laschet J., Varthaman A., Fornasa G., Compain C., Gaston A.-T., Clement M., Dussiot M., Levillain O., Graff-Dubois S., Nicoletti A. (2010). Macrophage Plasticity in Experimental Atherosclerosis. PLoS ONE.

[44] Luo L., Zhou W.-H., Cai J.-J., Feng M., Zhou M., Hu S.-P., Xu J., Ji L.-D. (2017). Gene Expression Profiling Identifies Downregulation of the Neurotrophin-MAPK Signaling Pathway in Female Diabetic Peripheral Neuropathy Patients. J. Diabetes Res..

[45] Steenman M., Espitia O., Maurel B., Guyomarch B., Heymann M.-F., Pistorius M.-A., Ory B., Heymann D., Houlgatte R., Gouëffic Y. (2018). Identification of Genomic Differences among Peripheral Arterial Beds in Atherosclerotic and Healthy Arteries. Sci. Rep..

[46] Qian Y., Xiong S., Li L., Sun Z., Zhang L., Yuan W., Cai H., Feng G., Wang X., Yao H. (2024). Spatial Multiomics Atlas Reveals Smooth Muscle Phenotypic Transformation and Metabolic Reprogramming in Diabetic Macroangiopathy. Cardiovasc. Diabetol..

