# Supplementary material for: Integrative Multi-Omics and Machine Learning Reveal Shared Biomarkers in Type 2 Diabetes and Atherosclerosis
Source: Int J Mol Sci. 2025 Dec 22;27(1):136. doi: 10.3390/ijms27010136 (PMC12786049; doi:10.3390/ijms27010136)

Figure S1. Single-cell RNA-seq quality control before filtering.
Violin plots of gene number (nFeature_RNA), UMI counts (nCount_RNA), mitochondrial percentage (percent.mito), and hemoglobin transcript percentage (percent.HB).

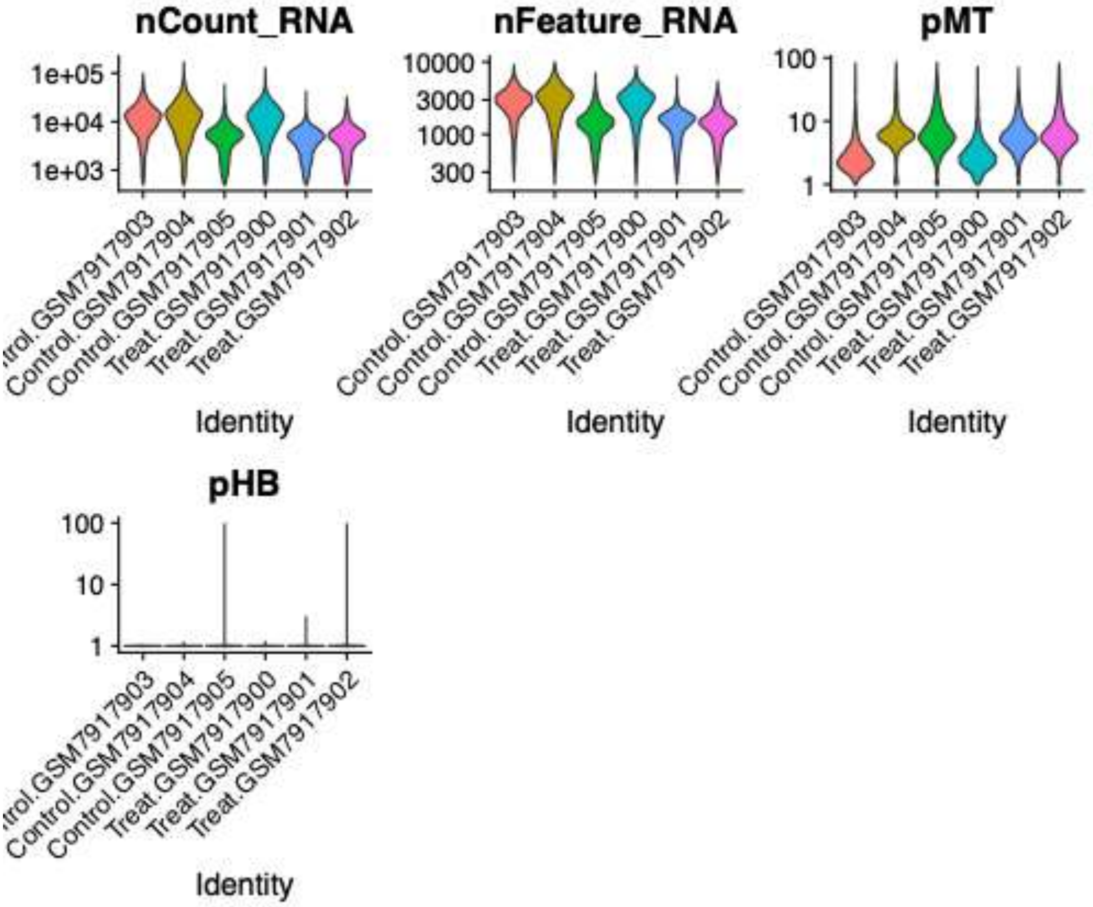

Figure S2. Single-cell RNA-seq quality control after filtering.
Violin plots of gene number (nFeature_RNA), UMI counts (nCount_RNA), mitochondrial percentage (percent.mito), and hemoglobin transcript percentage (percent.HB).

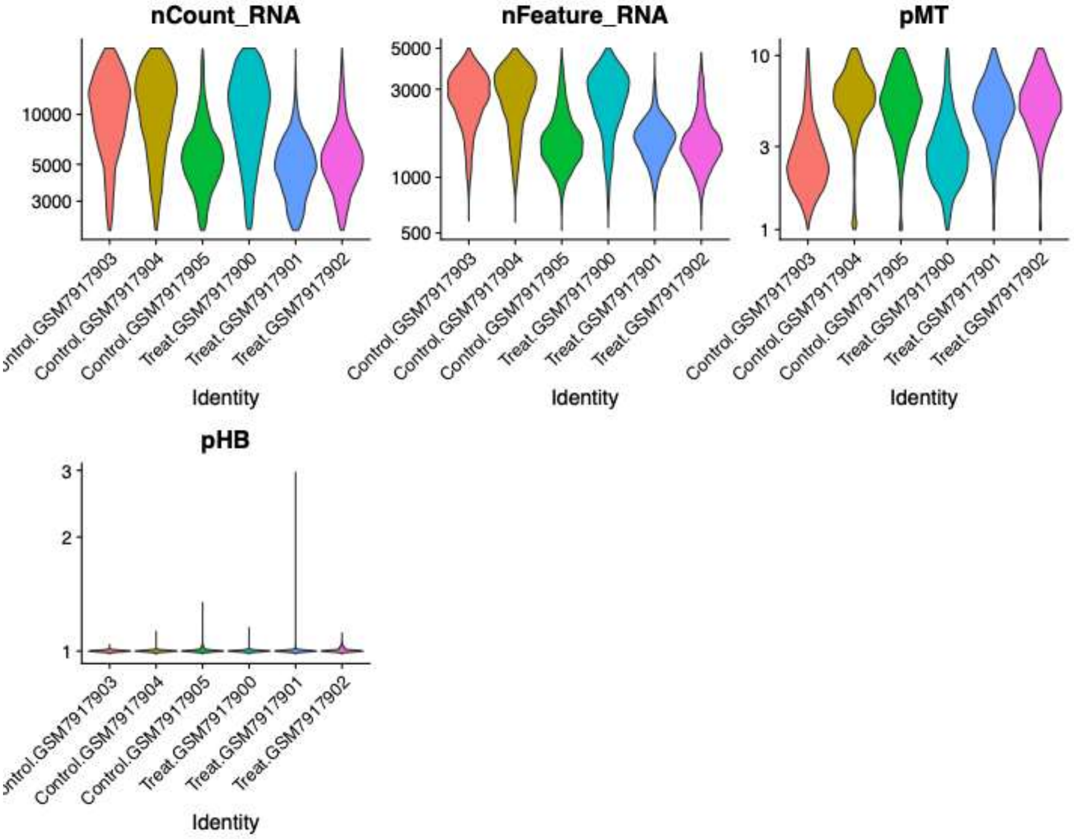

Figure S3. Unsupervised UMAP clustering.
Two-dimensional UMAP embedding of the post-QC cells colored by unsupervised clusters, yielding 21 clusters (clusters 0–20).

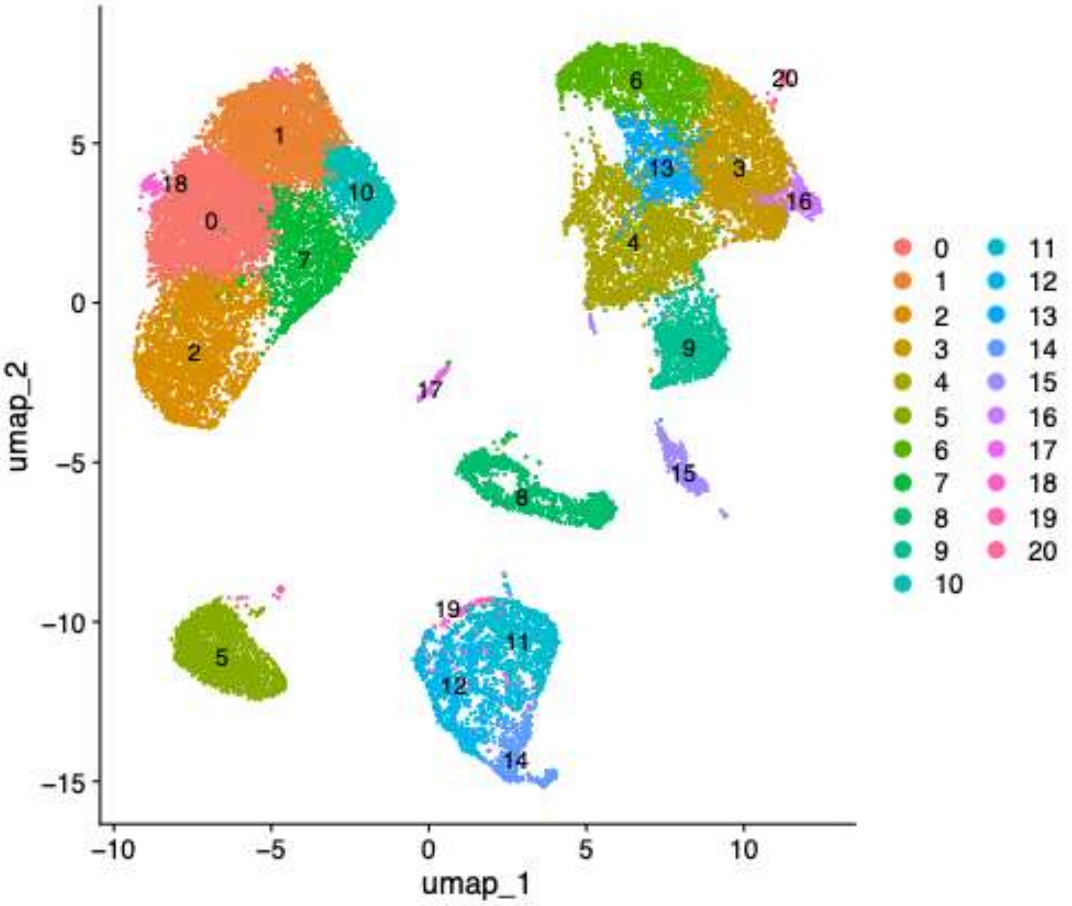

Supplement: Supplementary file 1 [file ijms-27-00136-s001.zip › Supplementary Figure.pdf]
